# Limbal Stem Cell Deficiency and Treatment with Stem Cell Transplantation

**DOI:** 10.4274/tjo.72593

**Published:** 2017-10-27

**Authors:** Özlem Barut Selver, Ayşe Yağcı, Sait Eğrilmez, Mehmet Gürdal, Melis Palamar, Türker Çavuşoğlu, Utku Ateş, Ali Veral, Çağrı Güven, Jose Mario Wolosin

**Affiliations:** 1 Ege University Faculty of Medicine, Department of Ophthalmology, İzmir, Turkey; 2 Ege University Faculty of Medicine, Department of Medical Biochemistry, İzmir, Turkey; 3 Ege University Faculty of Medicine, Department of Histology and Embriology, İzmir, Turkey; 4 İstanbul Bilim University Faculty of Medicine, Department of Histology and Embryology, İstanbul, Turkey; 5 Ege University Faculty of Medicine, Department of Pathology, İzmir, Turkey; 6 Ege University Faculty of Medicine, Department of Gynecology and Obstetrics, İzmir, Turkey; 7 Icahn Faculty of Medicine at Mount Sinai, Department of Ophthalmology and Black Family Stem Cell Institute, New York, USA

**Keywords:** Limbal stem cell deficiency, cultured cells, stem cell transplantation

## Abstract

The cornea is the outermost tissue of the eye and it must be transparent for the maintenance of good visual function. The superficial epithelium of the cornea, which is renewed continuously by corneal stem cells, plays a critical role in the permanence of this transparency. These stem cells are localized at the cornea-conjunctival transition zone, referred to as the limbus. When this zone is affected/destroyed, limbal stem cell deficiency ensues. Loss of limbal stem cell function allows colonization of the corneal surface by conjunctival epithelium. Over 6 million people worldwide are affected by corneal blindness, and limbal stem cell deficiency is one of the main causes. Fortunately, it is becoming possible to recover vision by autologous transplantation of limbal cells obtained from the contralateral eye in unilateral cases. Due to the potential risks to the donor eye, only a small amount of tissue can be obtained, in which only 1-2% of the limbal epithelial cells are actually limbal stem cells. Vigorous attempts are being made to expand limbal stem cells in culture to preserve or even enrich the stem cell population. *Ex vivo* expanded limbal stem cell treatment in limbal stem cell deficiency was first reported in 1997. In the 20 years since, various protocols have been developed for the cultivation of limbal epithelial cells. It is still not clear which method promotes effective stem cell viability and this remains a subject of ongoing research. The most preferred technique for limbal cell culture is the explant culture model. In this approach, a small donor eye limbal biopsy is placed as an explant onto a biocompatible substrate (preferably human amniotic membrane) for expansion. The outgrowth (cultivated limbal epithelial cells) is then surgically transferred to the recipient eye.

Due to changing regulations concerning cell-based therapy, the implementation of cultivated limbal epithelial transplantation in accordance with Good Laboratory Practice using xenobiotic-free systems is becoming widely accepted both in Turkey and worldwide.

## INTRODUCTION

### Limbal Stem Cell Deficiency

**Figure 1:**
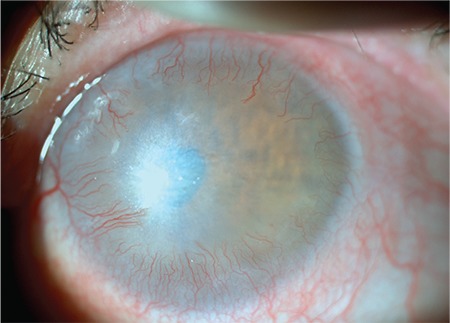
Photograph of a patient with limbal stem cell deficiency caused by chemical injury (acetone) showing conjunctivalization and marked vascularization advancing toward the central cornea

Limbal stem cell deficiency (LSCD) is a complex pathology with a multifactorial etiology, in which the cornea partially or completely loses its regenerative ability.^1^ Stem cell loss resulting from severe damage to the limbal zone leads to permanent corneal epithelial defects and vision loss due to conjunctivalization (Figure 1).^2^

### Etiology

The circumstances that lead to LSCD are divided into two main groups, primary causes and secondary causes (Table 1).

**Figure 2:**
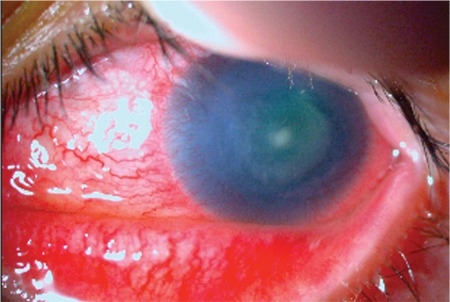
A patient with aniridia exhibits signs of limbal stem cell deficiency

Clinically, secondary causes are encountered more frequently than primary causes, in which genetic factors play a role in the etiology (e.g. aniridia, Figure 2).^3,4^

### Signs and Symptoms

LSCD has nonspecific symptoms including reduced visual acuity, photophobia, epiphora, blepharospasm, redness associated with chronic inflammation, and recurring attacks of pain due to epitheliopathy.^4,5^

On slit-lamp examination, the corneal epithelium presents a dull and irregular reflex. Depending on the severity of LSCD, thick fibrovascular pannus formation, chronic keratitis, scarring, and calcification may occur. The cornea often exhibits abnormal fluorescein staining due to increased permeability resulting from corneal conjunctivalization.^4^

### Diagnosis

It is important to establish a definitive diagnosis in LSCD. Failure to do so may result in the patient undergoing cornea transplantation, which has poor outcomes in this disease.^6^

**Figure 3:**
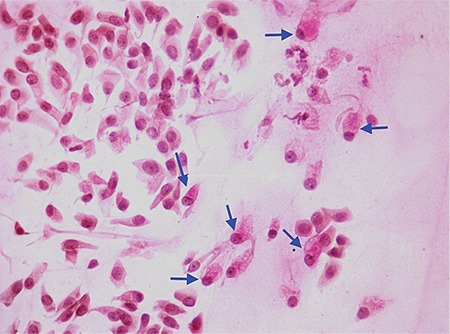
Impression cytology showing goblet cells (arrow) and squamous cells (PASx100)

Despite the many findings of LSCD, only conjunctivalization and goblet cell migration onto the corneal surface are important for diagnosis. Clinical signs of conjunctivalization are deterioration of the limbal palisades of Vogt or delayed fluorescein staining of the cornea. A primary diagnosis of conjunctivalization may be established by demonstrating the presence of goblet cells in the cornea using impression cytology (Figure 3).^1^

### Treatment Methods

There are several approaches to the treatment of LSCD. Among them are autologous and allograft limbal graft transplantations as well as cultivated limbal epithelial transplantation (CLET), which is becoming increasingly important.^6^

Autologous limbal grafts may be used in unilateral LSCD, with success rates of over 80% reported in the literature.^7,8^ Although not yet proven conclusively, the risk of LSCD development in the donor bed limits the ability to obtain sufficient donor tissue in autologous limbal grafts.^9^

Allograft is a treatment option in bilateral LSCD, but its success is limited due to the risk of immune reaction and allograft rejection.^5,6^ The expectations of long-term success with keratolimbal allografts are low, as success rates reported in the literature are around 50%.^10,11^

Although various surgical treatments are available, there is still no known reliable and effective treatment method for cases of severe LSCD, especially bilateral cases.^6^ For these reasons, the development of new treatment strategies such as limbal cell culture has become an inescapable necessity.^12,13,14,15,16,17^

The relatively new cell therapy methods recently introduced to clinical practice are still not fully understood with regard to their biological backgrounds. In particular, the characteristics of limbal stem cells and their microenvironments are among the main research topics in current limbal stem cell culture studies.^18^ Both established and developing techniques based on the transplantation of cultivated limbal stem or precursor cells have been shown to be useful for the treatment of serious ocular surface diseases, with success rates of approximately 73% for allogenic and 77% for autologous methods.^19^ However, the question of which technique is the most suitable remains to be answered.^12,16,20^

In 1997, Pellegrini et al.^13^ first reported that limbal stem cells can be cultivated on a “feeder layer” in the journal *Lancet*. Later, researchers used autologous limbal epithelial cells cultivated on human amniotic membrane (HAM) for cornea reconstruction.^17^ However, these methods were not sufficient for the formation of fully stratified and well-differentiated epithelial layers. As the markers specific to limbal stem cells could not yet be fully determined, it is not possible to adequately isolate them using physical techniques. Therefore, most studies have focused on characterization of the stem cell microenvironment which governs the genes that control limbal epithelial cell differentiation, and determining the signals required for the differentiation of the temporarily proliferating cells in limbal basal epithelium.^21,22,23,24^

CLET, which is becoming increasingly common, is the most advanced treatment method based on existing techniques. Better identification of stem cell properties and microenvironments and the discovery and development of new stem cell sources will allow us to develop ideal treatments and achieve better clinical results in the future.^3,25,26^

### Cultivated Limbal Epithelial Cells

**Tissue sources for cultivation:** The basic tissue sources for limbal epithelial cells are autologous limbal biopsy tissues, usually obtained from the patient’s healthy fellow eye in unilateral cases, and allogenic limbal biopsy tissues obtained from a living relative or from a cadaver in bilateral cases.^27,28,29^

**Culture method:** A limbal biopsy approximately 1x2 mm in size is obtained from the donor eye and transferred to the laboratory for culturing. There are two main culture methods: cell suspension culture and explant culture.^30^

**a. Suspension culture:** The biopsy sample taken from the limbal area is dissociated into individual cells using enzymes such as dispase, trypsin, and collagenase. These individual cells are grown in culture medium.^31,32^

**Figure 4:**
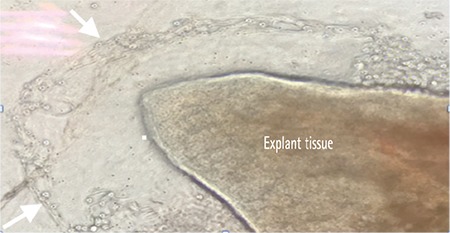
Inverted light microscopy (Olympus; CKX41) image showing explant biopsy tissue and cells (white arrows) proliferating from the edges of the tissue onto the human amniotic membrane

**b. Explant culture:** The explant tissue is placed on a substrate, allowing the proliferation and growth of cells onto the substrate surface (Figure 4).^33^

Several culture components are used in both of these cultivation methods to promote in vitro growth.

### Culture Components

**a. 3T3 cells:** 3T3 cells are mouse fibroblasts which are commonly used to enable the formation of epithelial layer in the culture. However, the use of these cells theoretically carries risks such as animal cell transplantation, infection, rejection, and microchimerism. Therefore, it is not typically preferred for clinical applications.^34^

**b. HAM:** HAM is a non-immunogenic biomembrane that has been applied clinically for many years to facilitate wound healing.35 Most researchers prefer to use deepithelialized HAM because morphological studies have shown that it better supports limbal epithelium growth.^14^ Although it is currently the most commonly used substrate in the clinical application of CLET, HAM has certain disadvantages including biological instability, lack of standardized preparation protocols, and a theoretical risk of infection.^19,36,37^

**c. Fibrin gel:** Fibrin gel is a bioabsorbant membrane composed of fibrinogen. Like HAM, a theoretical risk of infection cannot be ruled out because the fibrogen is human-derived.^38,39,40^

**Culture Medium:** During the limbal epithelium cell culture process, the culture medium is enriched with serum in order to facilitate proliferation and growth of the epithelium. Although bovine serum has traditionally been used for this purpose,^13,41,42^ recent legal regulations and the subsequent focus on xenobiotic-free culture systems have led to the increasing use of autologous serum.^43,44^

**Culture Duration:** The time required for culturing is about 2 weeks, but this period may be extended when using the air-lifting technique, in which the culture is exposed to the air-liquid interface in order create a multilayer epithelium.^19,39,40,44,45,46^

### Cultivated Limbal Epithelial Transplantation

**Figure 5:**
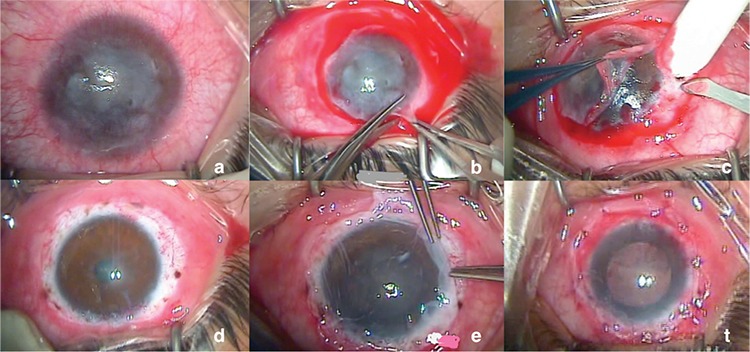
Stages of cultivated limbal epithelial transplantation

**a. Preparation of the ocular surface:** Prior to CLET, the eye with LSCD (Figure 5a) undergoes peritomy (Figure 5b), the fibrovascular pannus tissue is cleared from the ocular surface (Figure 5c), and superficial keratectomy is performed when necessary. After achieving hemostasis via cauterization, the ocular surface is ready for CLET (Figure 5d).

**b. CLET:** The cultivated limbal epithelium is transferred from the laboratory to the operating room and transplanted to the prepared ocular surface with the epithelial side up. It is usually fixed to the cornea or the episclera using 10-0 nylon sutures (Figure 5e).

**c. Application of a protective membrane/contact lens:** Following transplantation, usually a protective HAM or sometimes a bandage lens is placed to prevent the transplanted cells from being adversely effected by eyelid movements. If HAM is used, it is fixed to the conjunctiva using 8-0 vicryl sutures (Figure 5f).^19,28,47^

The methods employed in published prospective studies evaluating the long-term outcomes of CLET in LSCD are summarized in Table 2.

### New Regulations

The use of cell therapies is becoming increasingly common worldwide, thus necessitating the development of new standards and protocols.^48^ The need for xenobiotic-free methods in CLET emerged due to the risks associated with the use of animal-derived components, including the transmission of a number of diseases (e.g. prion disease), the development of tumorigenic effects, and immune reactions.^25,34,49,50^ Furthermore, in order to protect public health when transplanting processed medical products into humans, these techniques must be performed under good laboratory practices, a requirement which has been passed into law in many European countries.^39,43,51^

In Turkey, the Regulation on Human Tissue and Cells and the Quality and Reliability of Related Health Centers issued by the Ministry of Health of the Republic of Turkey was published in the official journal dated October 27, 2010.^52^

## CONCLUSION

The dynamic equilibria that ensure the continuity of epithelialization are important in the processes of ocular surface healing and homeostatic regulation. LSCD, which arises as a result of the disturbance of these dynamic balances, is a painful and tiring condition which impairs vision and reduces quality of life. Multidisciplinary basic and clinical studies are ongoing to develop more effective treatments for LSCD. Surgical success is expected to increase with the development of targeted and effective methods that suppress inflammation by restoring ocular surface homeostasis, enhancing regeneration, and inhibiting neovascularization. Advances in the identification and implementation of ideal scaffolds for stem cell proliferation and in our understanding of the limbal microenvironment will lead to increases in the quantity and quality of the stem cell populations used in CLET, which will in turn result in improved healing and graft success. For ocular surface restoration, especially in bilateral cases lacking an autologous cell source, further development of tissue engineering techniques and the ability to transform non-limbal stem cell sources (e.g. induced pluripotent stem cells) into limbal stem cells by modifying their models of biological behavior may open new horizons in the treatment of this complex pathology.

## Figures and Tables

**Table 1:**
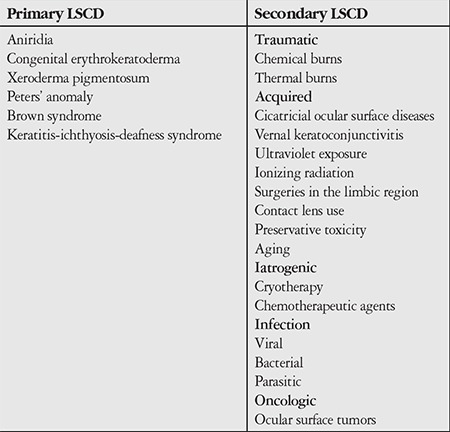
Classification of the causes of limbal stem cell deficiency

**Table 2:**
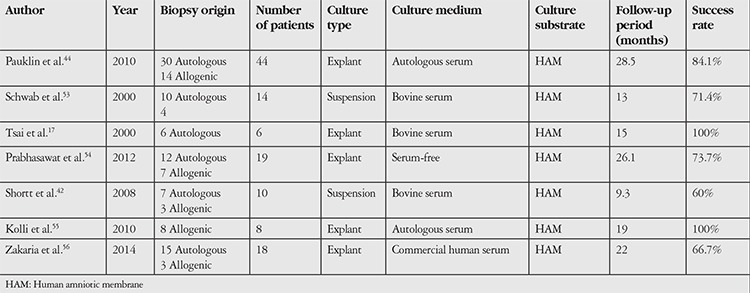
Features of the cell culture methods utilized in published prospective studies of the long-term outcomes of cultured limbal epithelial transplant
